# Downregulation of ARHGDIA contributes to human glioma progression through activation of Rho GTPase signaling pathway

**DOI:** 10.1007/s13277-016-5374-6

**Published:** 2016-10-10

**Authors:** Weiliang Lu, Xixi Wang, Jingjing Liu, Yu He, Ziwei Liang, Zijing Xia, Ying Cai, Liangxue Zhou, Hongxia Zhu, Shufang Liang

**Affiliations:** 1 State Key Laboratory of Biotherapy and Cancer Center, West China Hospital, Sichuan University and Collaborative Innovation Center for Biotherapy, No.17, 3rd Section of People’s South Road, Chengdu, 610041 China; 2 Department of Neurosurgery, West China Hospital, Sichuan University, Chengdu, 610041 Sichuan China; 3 Laboratory of Cell and Molecular Biology & State Key Laboratory of Molecular Oncology, Cancer Institute & Cancer Hospital, Chinese Academy of Medical Sciences, Beijing, 100034 China

**Keywords:** ARHGDIA, Glioma, Downregulation, Rho GTPase

## Abstract

The protein ARHGDIA has been found to play distinct roles in cancer progression for several tumors. However, it remains elusive whether and how ARHGDIA plays functions in human glioma. In this study, we discovered that ARHGDIA is much downregulated in human glioma; meanwhile, its expression negatively correlates with glioma malignancy and positively relates to prognosis of glioma patients. It has independent predictive value of ARHGDIA expression level for overall survival of human glioma patients. Glioma patients with ARHGDIA-positive expression have a longer overall survival time than ARHGDIA-negative patients. Knockdown of ARHGDIA promotes cell proliferation, cell cycle progression, and cell migration due to the activation of Rho GTPases (Rac1, Cdc42, and RhoA) and Akt phosphorylation, whereas overexpression of ARHGDIA suppresses cell growth, cell cycle progression, and cell migration. ARHGDIA is a potential prognostic marker and therapeutic target for human glioma.

## Introduction

Glioma is the most common brain tumor, accounting for about 45 % of all brain tumors [1]. The World Health Organization (WHO) classifies gliomas based on the different histological tumor types (astrocytic, oligodendroglial, mixed oligoastrocytic, and ependymal glioma), as well as malignancy grades (I, II, III, and IV) [2]. The present treatment options of glioma contain surgery, chemotherapy, and radiotherapy or the combination treatment of these methods, but the prognosis of malignant glioma remains very poor. The median survival is only 12 to 15 months for patients with glioblastoma and 2 to 5 years for patients with anaplastic glioma in the USA [3]. So, it is necessary to find biomarkers for early diagnosis and effective therapeutic targets to improve the prognosis for patients with human glioma [4].

The Rho GDP-dissociation inhibitors ARHGDIs (also named RhoGDIs) are important regulators of the Rho family of small GTPases which involves in cancer occurrence [5]. ARHGDIs take part in several biological processes during tumorigenesis and cancer progression. For example, the expression of ARHGDIs is altered in a variety of cancers, including breast cancer [6] and hepatocellular carcinoma [7]. Several novel therapeutic strategies are proposed for intervening in ARHGDI signaling [8]. These reports indicate that ARHGDI signaling may be targets for cancer therapy.

ARHGDIA is one member of ARHGDIs, which is ubiquitously expressed and interacts with several Rho GTPases, mainly including RhoA, Rac1, and Cdc42 [9]. As a regulator of Rho GTPase activity, ARHGDIA has attracted increasing attentions. Previous studies have indicated the aberrant expression of ARHGDIA is associated with cancers [6, 7], whereas there is no research in detail on glioma. So our investigation aim is to explore ARHGDIA functions in glioma development. Our analyses reveal that ARHGDIA is frequently downregulated in human glioma tissues and it is significantly associated with tumor malignancy degree.

## Materials and methods

### Glioma patients and tissue samples

This study was approved by the Institutional Ethics Committee of State Key Laboratory of Biotherapy, West China Hospital of Sichuan University. A total of 73 glioma patients were enrolled in our study. The clinical information of patients was summarized in Table 2. The patients did not receive any preoperative cancer treatment, and their follow-up data were available. They were followed-up since the surgical treatment until May 2015, with a median follow-up of 20.4 months (range 0.2–68 months). During the follow-up, patients were monitored every 2–3 months by clinic interview or phone call.

Seventy-three pairs of human glioma tissues (HGTs) and patients’ autologous para-cancerous brain tissues (PBTs) were surgically resected to collect with patient’s informed consent in West China Hospital, Sichuan University (Chengdu, P. R. China).

### Immunohistochemical analysis

The tissues paraformaldehyde-fixed and paraffin-embedded were cut into sections of 5 μm thickness for hematoxylin-eosin (HE) and immunohistochemistry (IHC) analysis mainly according to our previous protocols [10]. The anti-ARHGDIA antibody (Santa Cruz Biotechnology, 1:100) was used to detect the protein expression in human glioma and non-cancer tissues. Finally, the tissue slices were visualized by the 3,3-diaminobenzidine solution and nuclei were slightly counterstained with hematoxylin. Substitution of the primary antibody with phosphate-buffered saline was served as a control for IHC. The intensity and percentage of positive cells were evaluated in at least five separate fields at ×400 magnification.

The staining intensity was scored as 0 (negative), 1 (weak), 2 (moderate), or 3 (strong). The extent of staining was scored based on the percentage of positive tumor cells: 0 (negative), 1 (1–25 %), 2 (26–50 %), 3 (51–75 %), and 4 (76–100 %) [11, 12]. The final score of each sample was assessed by summarization of the results of the intensity and extent of staining. Therefore, each case was considered negative if the final score was 0 (−) and positive if the final score was 1–2(weak, +), more than 2 (strong, ++), respectively. The scoring was determined independently by two senior pathologists.

### Association analysis of ARHGDIA expression with glioma clinical information

The relationship between ARHGDIA expressions with glioma clinical information was assessed based on IHC data of glioma tissues using Pearson’s χ^2^ test. The clinical information included glioma patient’s gender, age, tumor grades (seen in the Table 2), and patient prognosis. Among 73 glioma patients, only 37 patients whose follow-up time was more than 5 years, and the follow-up time of other 36 glioma patients was less than 5 years but all more than 3 years. The patient overall survival (OS) was evaluated using the Kaplan-Meier method. The 73 glioma patients were grouped into two groups based on the protein expression level, including ARHGDIA-negative expression (*n* = 32) and ARHGDIA-positive expression (*n* = 41). The group differences were assessed using the log-rank test.

### Cell culture

Human glioma cell lines H4 and U87 were ordered from American Type Culture Collection (Manassas, VA), and U251 were ordered from the Type Culture Collection of the Chinese Academy of Sciences (Shanghai, China). Cells were cultured in DMEM medium containing 10 % fetal bovine serum (FBS) (16000-044, Gibco), with 100 U/ml penicillin and 100μg/ml streptomycin. Cells were incubated in 37 °C with 5 % CO2 and 95 % air.

### ARHGDIA-specific siRNAs, expression plasmid, and cell transfection

In order to reduce the off-target effects of single siRNA, three different siRNAs against ARHGDIA had been designed and synthesized to perform the ARHGDIA knockdown experiments. One of siRNAs, siRNA1 against ARHGDIA, was synthesized based on one previous reported paper [13]. The other two siRNAs, siRNA2 and siRNA3, were designed according to our bioinformatics analysis and synthesized by the RiboBio company (RiboBio, Guangzhou, China). ARHGDIA-specific siRNA1 sequences were designed as follows: 5′-UCAAUCUUGACGCCUUUCC-3′. The siRNA2 and siRNA3 sequences were respectively designed as following: (siRNA2) 5′-GAGCACTCGGTCAACTACA-3’and (siRNA3) 5′-GGTGTGGAGTACCGGATAA-3′. The non-targeting control siRNA oligonucleotides were 5′-UUC UCC GAA CGU GUC ACG U-3.

In order to observe cell growth and Akt signaling under ARHGDIA knockdown, 100 nM ARHGDIA-specific siRNA was respectively transiently transfected into glioma cells for one well of a 6-well plate for 48 h culture using the INTERFERin transfection reagent (Polyplus Transfection).

The ARHGDIA cDNA (gi 669033301) was cloned into the expression vector pcDNA3.1-HA to obtain the recombinant plasmid pHA-ARHGDIA. The overexpression of ARHGDIA by transfection of pHA-ARHGDIA plasmids into U87 cells was performed to detect its effects on cell growth and migration. Two-microgram plasmids were transfected for each well of a 6-well plate with the reagent Lipofectamine 2000 (Cat. 11668-019, Life Technologies).

### Quantitative RT-PCR

To compare endogenous gene expression in HGTs versus PBTs, RNA samples were prepared from HGTs or PBTs using TRIZOL reagent (Cat. #15596-026, Invitrogen). First-strand complementary DNA (cDNA) synthesis was carried out with the cDNA synthesis kit (Cat. #170-8891, Bio-Rad). PCR reaction was carried out with first-strand cDNA and one set of specific primers. For each primer set, two or three cycle numbers were tested to confirm that PCR product accumulates within a linear range. The GAPDH was amplified as a control marker with specific primers. The relative RNA expression was calculated with the comparative CT method, which was normalized to the internal references. The RT-PCR primers for ARHGDIA were designed as follows: forward primer 5′-CCTCACACTGCCCCAGAGGAT-3′ and reverse primer 5′-GCGCACTTCTGAGCAGGAGT-3′ [14]. The forward primer 5′- TGG AAG GAC TCA TGA CCA CA-3′ and reverse primer 5′- TTC AGC TCA GGG ATG ACC TT-3′ for the control GAPDH.

### Western blot

Cell pellets were harvested after being transfected with ARHGDIA-specific siRNAs or the pHA-ARHGDIA plasmids for 48 h. Cells were lysed to extract proteins with RIPA buffer (50 mM Tris base,1.0 mM EDTA, 150 mM NaCl, 0.1 % SDS, 1 % Triton X-100, 1 % sodium deoxycholate, and 1 mM PMSF). Proteins were separated on 12 % SDS-PAGE, transferred to polyvinylidene difluoride (PVDF) membrane to incubate with rabbit anti-ARHGDIA antibody (1:100, Santa Cruz Biotechnology) at 4 °C overnight. A secondary antibody incubation was performed with horseradish peroxidase (HRP)-tagged anti-goat (Santa Cruz) or HRP tagged anti-rabbit (Invitrogen) antibody. The PVDF membrane was re-probed with mouse anti-GAPDH antibody (Abcam) for normalization of signal. Detection was performed with Western blot reagent ECL (Amersham Biosciences).

### Pull-down analysis of active Cdc42/Rac1 and RhoA

The pull-down procedures were performed based on the commercial antibody against the active form of Cdc42, Rac1, and RhoA (MuCyte Biotechnology, Nanjing, China). Cell pellets from U87 cells transfected with ARHGDIA-specific siRNA1 or pHA-ARHGDIA plasmids for 48 h were collected and resolved in 1 ml of ice-cold 1 × assay/lysis buffer for 10 min. Protein supernatant was obtained by centrifugation with 14,000×*g* for 10 min at 4 °C. Fifty microliters slurry of glutathione resin was washed with 1 × assay/lysis buffer, and 60 μl of GST-RBD (Rho-binding domain ) or 20 μl GST-PBD(p21-binding domain) was added to bind with the resin on ice for pull-down active RhoA, Rac1, or Cdc42, respectively. Then 1 mL protein solution was added to incubate with the antibody-combined resin at 4 °C for 1 h with gentle agitation. The supernatant was removed by 7200×*g* centrifugation for 1 min, and the resin was washed three times with 0.5 ml of 1 × assay/lysis buffer, suspended in 30 μl of 2× reducing SDS-PAGE sample buffer to pull down the target proteins by boiling for 5 min. The sample was separated on 12 % SDS-PAGE to detect the target proteins by Western blot. The first antibodies included rabbit monoclonal antibody against RhoA (1:1000, Sion Biological), mouse monoclonal antibody against Cdc42 (1:1000, Abcam), and mouse monoclonal against Rac1 (1:1000; Abcam).

### Cell proliferation

Cell proliferation was measured using the Cell Counting Kit-8 (CCK-8) assay (cat.ZP328-3, ZOMANIO). Three thousand cells were plated into each well of a 96-well plate after transfection with pHA-ARHGDIA plasmids or ARHGDIA-specific siRNA1 for 48 h, in which 10 μl CCK-8 reagents was added to 90 μl of culture medium. Cells were subsequently incubated for 2 h at 37 °C and the absorbance was measured at 450 nm on Multiskan MK3 (Thermo Scientific). Three independent experiments were performed.

### Flow cytometry

For cell cycle analysis, cells were harvested after siRNA1 treatment for 48 h, fixed in 70 % ethanol on ice and stained with propidium iodide in phosphate-buffered saline containing RNase for 15 min at 37 °C in the dark. The DNA contents were analyzed by flow cytometry (NovoCyte, ACEA Biosciences).

### In vitro migration assays

Cell migration was performed using our previous method [11]. The 1 × 10^4^ cells were added into the upper chamber of the insert with the non-coated membrane (Millipore, 8-mm pore size). Cells were plated in serum-free medium, and medium containing 10 % FBS in the lower chamber served as chemo-attractant. After 24 h of incubation, cells that did not migrate through pores were carefully wiped out with cotton swab. Cells on the lower surface of the membrane were fixed with methanol and stained with Giemsa (cat. C0121, Beyotime). Images were captured using an inverted microscope (Olympus), and the migrated cells were counted manually. Each experiment was performed in triplicates.

### Statistical analysis

Statistical analysis was performed and values were expressed as the mean ± standard deviation. The differences between groups were analyzed using Student’s *t* test (only two groups) or one-way analysis of variance (more than two groups were compared). *P* < 0.05 was considered statistically significant.

## Results

### ARHGDIA downregulation in glioma tissues

**Fig. 1:**
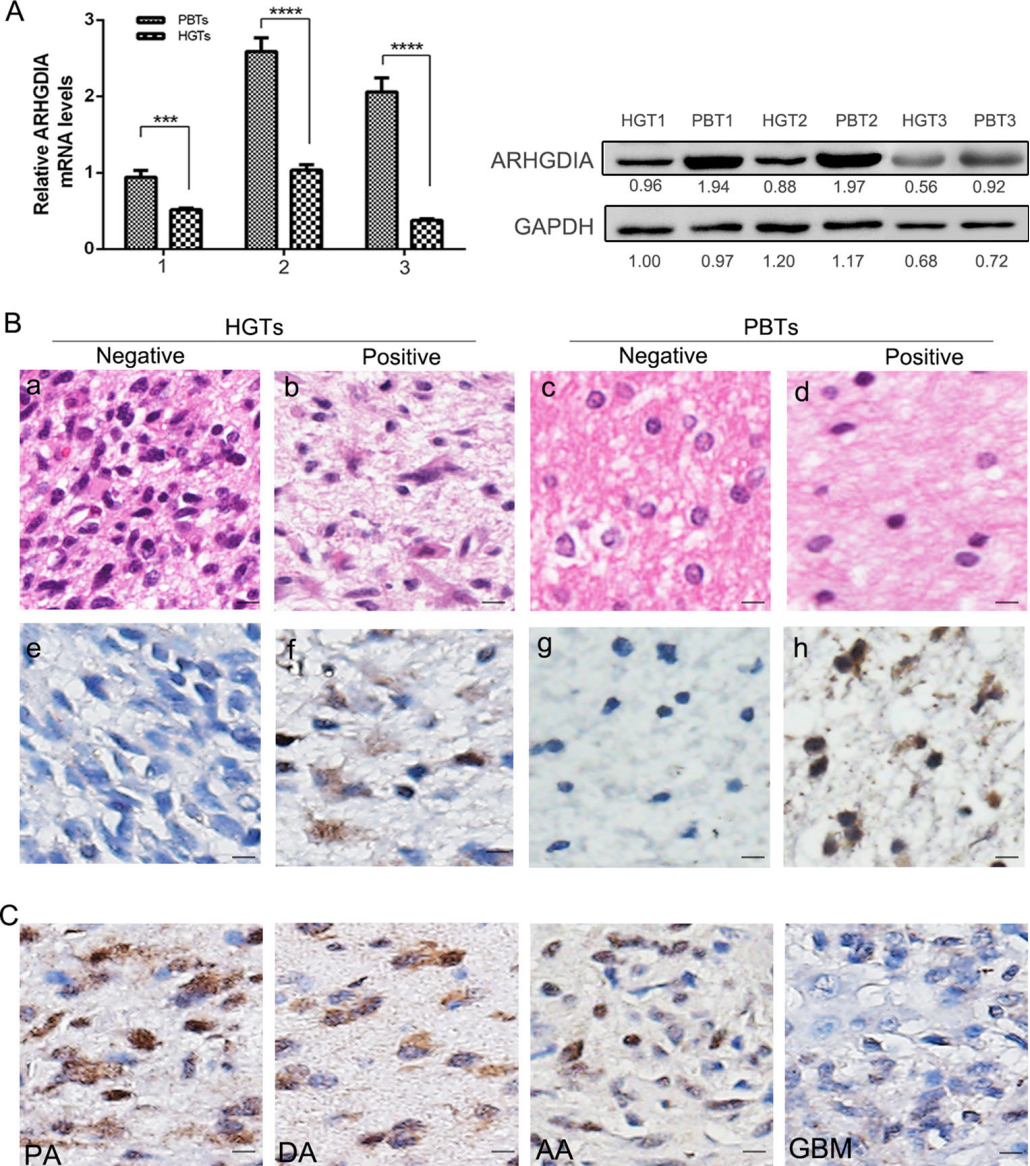
ARHGDIA is downregulated in human glioma. **A** ARHGDIA is detected in three randomly chosen HGTs via real-time PCR and western blot analysis. The transcript levels were normalized to GAPDH. Data are presented as the mean and standard deviation (SD) of three independent experiments. **B** Immunohistochemical staining of ARHGDIA in HGTs and PBTs. Tissue hematoxylin-eosin staining (*a*–*d*); ARHGDIA-negative (*e*) and ARHGDIA-positive expression (*f*) in HGTs, ARHGDIA-negative (*g*) and ARHGDIA-positive expression (*h*) in PBTs. **C** ARHGDIA expression in different histological glioma tissues. *PA:* pilocytic astrocytoma, *DA:* diffuse astrocytoma, *AA*: anaplastic astrocytoma, *GBM*: glioblastoma. Scale bar represents 50 μm (original magnification × 400). *HGTs*: human glioma tissues; *PBTs*: para-cancerous brain tissues. ***means *p* < 0.01, ****means *p* < 0.001

The low expression status of ARHGDIA at mRNA and protein levels was detected in HGTs. Compared with the expression level in PBTs, ARHGDIA was greatly decreased in glioma tissues both at mRNA and protein levels (Fig. 1A). Moreover, the decreased expression of ARHGDIA was validated in 73 cases of HGTs compared with 13 PBTs by IHC (Table 1). As results, more than one thirds of human glioma tissues (*n* = 25, 34.2 %) had ARHGDIA-negative expression, and other 48 cases (65.8 %, 48/73) showed weak expression of ARHGDIA in cytoplasm (Fig. 1B-f ), with mean staining scores 1.396 ± 0.08. While in 84.6 % (11/13) PBTs, ARHGDIA was detected with a higher expression level with an average staining score 2.375 ± 0.42 (Fig. 1B-h). Generally, a lower expression of ARHGDIA widely exists in gliomas than that in noncancerous brain tissues (*p* < 0.05).

**Table 1:** ARHGDIA immunoreactivity between HGTs and PBTs

Immuno -reactivity	HGTs (*n* = 73)		PBTs (*n* = 13)		*p* value
	Percentage	Score	Percentage	Score	
Negative	34.2 %(25/73)	0 (−)	15.4 %(2/13)	0 (−)	
Positive	65.8 %(48/73)	1.396 ± 0.08 (+)	84.6 %(11/13)	2.375 ± 0.42 (++)	*p* < 0.05

*P* value was calculated the difference between ARHGDIA-positive HGTs and PBTs using Student’s *t* test. *P* < 0.05 was considered statistically significant

*HGTs*: human glioma tissues, *PBTs*: para-cancerous brain tissues

−: negative; +: weak expression; ++: strong expression

### ARHGDIA downregulation correlates with tumor stage and patient survival

In order to evaluate whether ARHGDIA is a potential diagnosis or prognosis factor in clinical test, we analyzed the relationship between the expression of ARHGDIA and the clinicopathologic features of human glioma patients. The expression levels of ARHGDIA in glioma exhibit a tumor pathological grade-dependent pattern (Table 2, *p* = 0.048). And a significantly relative stronger ARHGDIA exists in high differentiated glioma (TNM stage I and II) than in low differentiation tumors (TNM stage III and IV) (Table 2). Moreover, ARHGDIA expression differs in different histological gliomas, including pilocytic astrocytoma, diffuse astrocytoma, anaplastic astrocytoma, and glioblastoma (Fig. 1C). But the expression level of this protein has no relationship with patient’s gender and age (Table 2).

**Table 2:** Associations of ARHGDIA expression with the clinical features of glioma patients (*n* = 73)

Clinicopathologic variables	ARHGDIA expression		Average score	Expression level	*p* value
	Negative (*n* = 24)	Positive (*n* = 49)			
Gender					
Male	9	30	1.42 ± 0.10	+	0.081
Female	15	19	1.35 ± 0.15	+	
Age, (years)					
≤56	10	31	1.46 ± 0.11	+	0.131
>56	14	18	1.28 ± 0.11	+	
TNM stage					
I–II	11	9	1.67 ± 0.23	+	0.048
III–IV	12	33	1.27 ± 0.08	+	
Unknown	1	7			

+: ARHGDIA staining was scored 1–2 (weak expression)

**Fig. 2:**
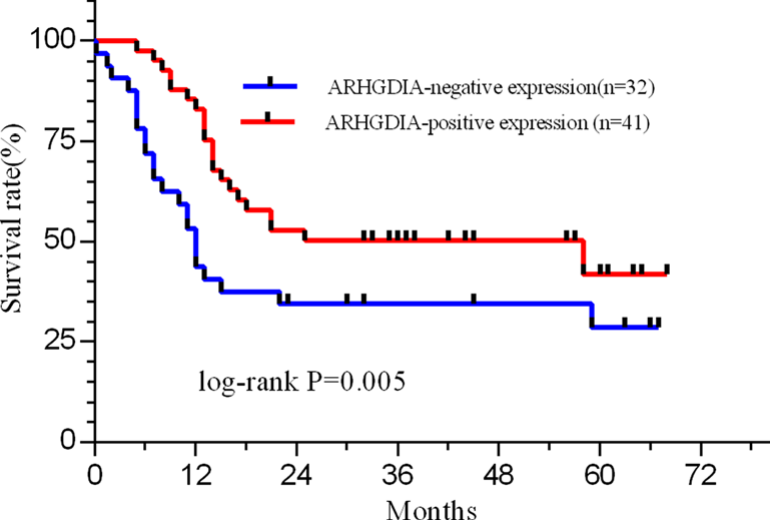
Association of ARHGDIA expression and overall survival of glioma patients. The overall survival has highly significant differences between ARHGDIA-positive patients (*n* = 41) and ARHGIDA-negative expresses (*n* = 32) (*p* < 0.05 by the log-rank test). The 1-, 3-, and 5-year OS rates of the patients with low level were 39.8, 30.1, and 26.7 %, respectively, which were significantly lower than those with high level group (78.6, 52.4, and 44.8 %, respectively; *p* = 0.005). Patients with low level of ARHGDIA have a worse postoperative overall survival

Furthermore, to determine the relationship between protein expression and OS of patients, 73 glioma patients were grouped into two groups, including ARHGDIA negative (*n* = 32) and positive (*n* = 41) expression. The ARHGDIA expression level is associated with prognosis of 74 glioma patients whose follow-up time more than 3 years. The Kaplan–Meier estimates showed significant differences in OS rates between ARHGDIA-negative patients and those with ARHGDIA-positive expression (*P* = 0.005 by the log-rank test; Fig. 2). The 32 patients with ARHGDIA-negative expression had a worse postoperative OS compared to the 41 cases of ARHGDIA-positive group (*p* = 0.005). The median survival in 41 ARHGDIA-positive patients was 25.0 months, whereas the median survival of ARHGDIA-negative patients was just 11.0 months. On the other hand, the 1-, 3-, and 5-year OS rate of the patients with the protein negative expression was 39.8, 30.1, and 26.7 %, respectively. Whereas the 1-, 3-, and 5-year OS rate of patients with the protein positive expression was 78.6, 52.4, and 44.8 %, respectively. All these data mean that patients with ARHGDIA-positive expression have a longer overall survival. Generally, ARHGDIA expression level is associated with tumor grade, survival, and prognosis of glioma patients.

### ARHGDIA expression regulates cell proliferation and cell cycle

**Fig. 3:**
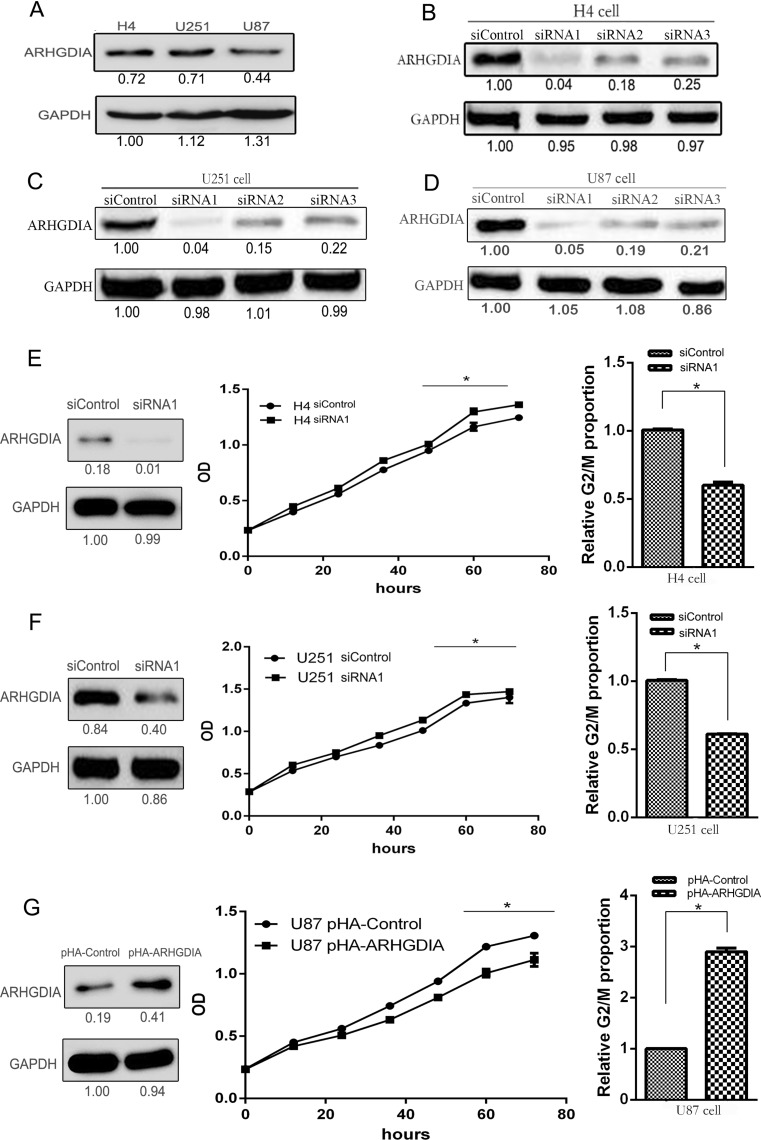
ARHGDIA downregulation promotes cell proliferation in vitro. **a** ARHGDIA expression in different human glioma cell lines. **b**–**d** The interference effect of three siRNAs in H4, U251, and U87 cells. **e**–**f** ARHGDIA knockdown in H4 (**e**) or U251 cells (**f**) by siRNA1 treatment for 48 h promotes cell proliferation by decreasing proportion of G2/M-phase cells (*p < 0.05). siControl: nontargeting control siRNA. **g** The overexpression of ARHGDIA in U87 cells with transfection of pHA-ARHGDIA plasmids for 48 h inhibits cell proliferation by increasing the proportion of G2/M-phase cells (*p < 0.05)

We detected the endogenous expression level of ARHGDIA in three different malignant glioma cell lines, including H4, U251 and U87 cells. The expression level of ARHGDIA in H4 and U251 has no obvious difference, but this protein is expressed in a relatively low level in U87 cells (Fig. 3a). We further explored this protein effects on cell growth and cell biological process by ARHGDIA knockdown in H4 cell, U251 cells and ARHGDIA overexpression in U87 cells.

The three different siRNAs had obvious interference effects for the target gene ARHGDIA expression in H4, U251, and U87 cells (Fig. 3b–d). The siRNA1 against ARHGDIA had the most effective inhibition for the protein. The inhibitory efficiency of 100 nM specific siRNA1 was up to 90 % at protein expression in these glioma cells. So we further measured cell growth responsive to siRNA1 treatment. ARHGDIA knockdown with siRNA1 significantly improved cell proliferation rate by decreasing cell proportion of G2/M-phase in H4 and U251 cells (Fig. 3e, f).

On the contrary, when the ARHGDIA protein is overexpressed to two times after being transfected with pHA-ARHGDIA plasmids in U87 cells, cell proliferation rate obviously decreased by increasing the proportion of G2/M-phase cells (Fig. 3g). The results suggest that ARHGDIA downregulation promotes glioma cell proliferation by regulating cell cycle distribution.

### ARHGDIA downregulation promotes glioma cell migration

**Fig. 4:**
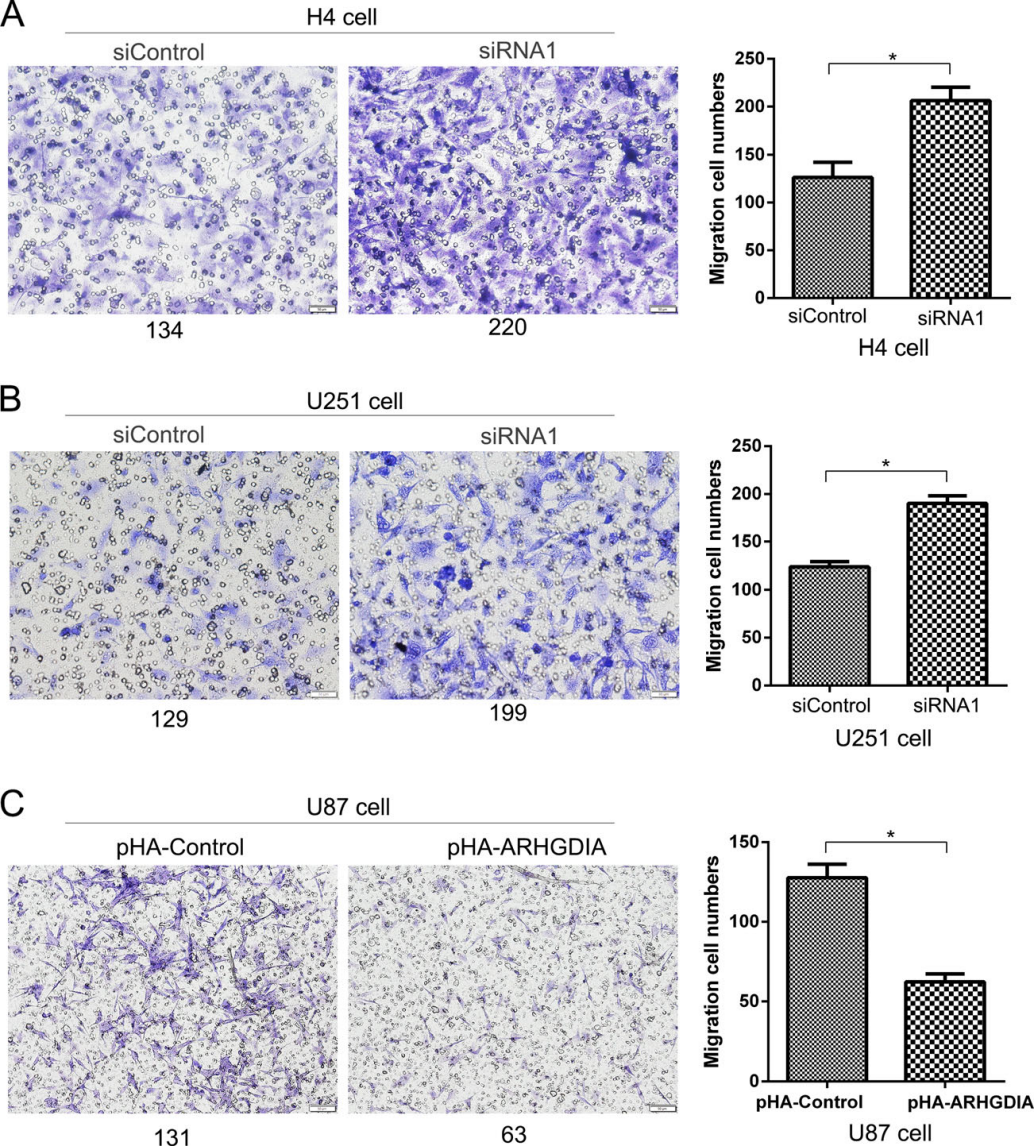
ARHGDIA knockdown promotes cell migration in H4 and U251 cells (**a**–**b**). While ARHGDIA overexpression in U87 cells inhibits cell migration (**c**). NC: nontargeting control siRNA or pHA empty vector. *p < 0.05

Next, cell migration was further analyzed to know if ARHGDIA has a crucial role in glioma cell migration ability. When ARHGDIA was knockdown by siRNA1 in H4 and U251 cells, the average number of migrated cells was significantly increased to near twofold in comparison with those by nonspecfic siRNA treatment (*P* < 0.05, Fig. 4a, b). This indicated that the migration potential of human glioma cells was markedly promoted after knockdown of ARHGDIA. Conversely, ARHGDIA overexpression in U87 cells resulted in low ability of cell migration (Fig. 4c).

In conclusion, the ARHGDIA downregulation may be an important contributor to cell migration, and the expression level of ARHGDIA affects the metastatic behavior of human glioma cell lines.

### Downregulation of ARHGDIA increases activity of Rho GTPases

**Fig. 5:**
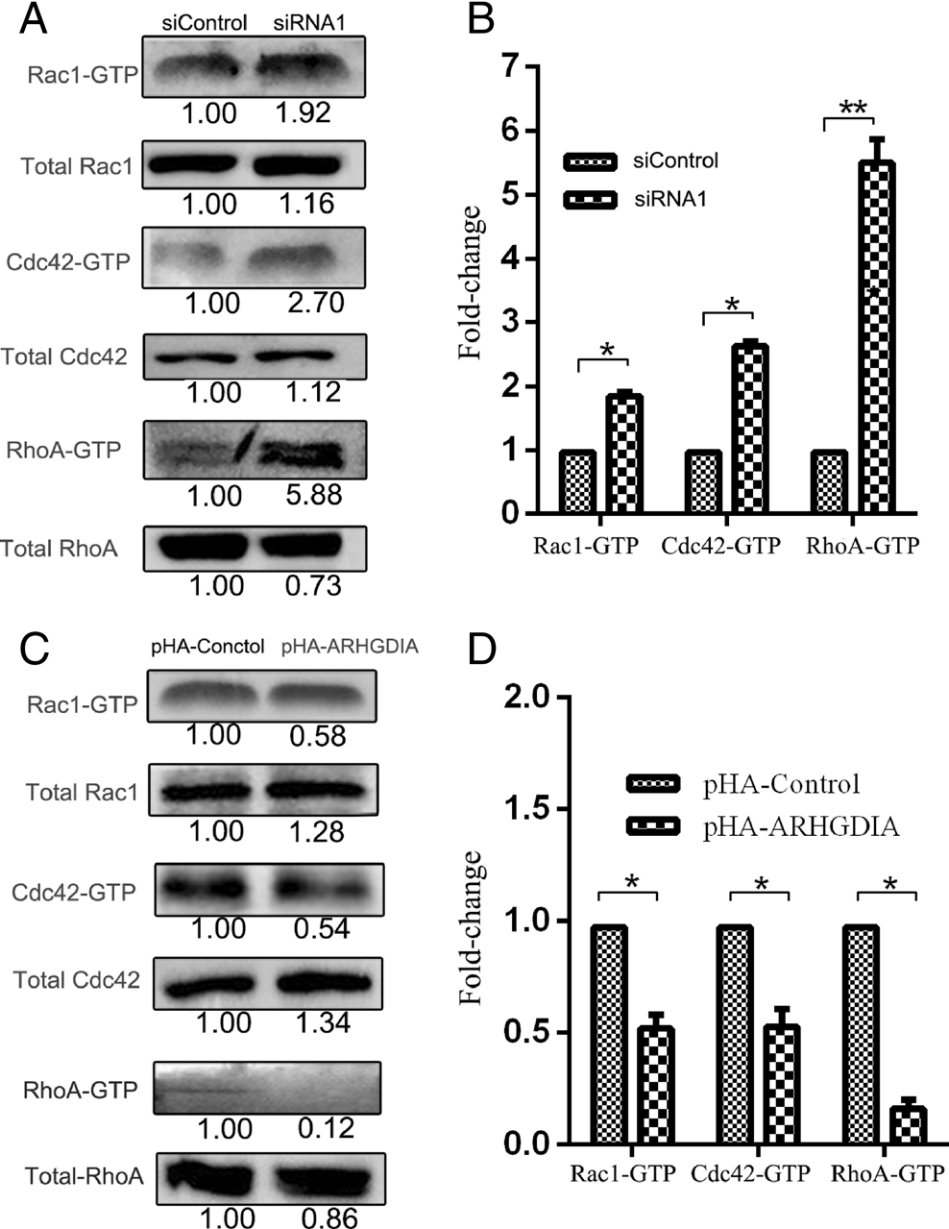
ARHGDIA knockdown (**a**) or overexpression (**c**) affects the GTPase activity of Rho family proteins. Rac1, Cdc42, and RhoA with GTP form represents the GTPase activity, which was analyzed from cell lysis after enrichment by pull-down. Total target proteins (Rac1, Cdc42, and RhoA) were present in whole cell lysate. The integrated density measurements were calculated based on the fold change of active-form protein versus total target protein. The gray value of control treatment (NC) was set as 1. The immunoblot quantitation of GTPase activity was shown in (**b**) and (**d**). *NC* nontargeting control siRNA or pHA empty vector. *p < 0.05, **p < 0.01

ARHGDIs regulate the cytosol-membrane cycling of the Rho GTPase, which has a major role in controlling Rho GTPase activity and function [5]. Due to the important role of ARHGDIA in the regulation of Rho GTPase, then how does its decreased expression regulate its effector proteins including Rac1/cdc42 and RhoA in glioma cells? Based on the above conclusion of lower ARHGDIA contribution to cell growth and cell migration, we conducted pull-down assays to determine the GTP status of Rac1/cdc42 and RhoA in U87 cells after being transfected with siRNA1 and pHA-ARHGDIA plasmids in triplicates. As expected, the lower expression of ARHGDIA significantly induced the activation of Rac1, Cdc42, and RhoA with GTP status in U87 cells treated with ARHGDIA siRNA1 (Fig. 5a). In response to ARHGDIA knockdown, the GTPase activity of Cdc42 and RhoA was averagely increased to 2.63- and 5.50-folds, respectively, and the Rac1 was also elevated to nearly twofolds (Fig. 5b). But the higher expression of ARHGDIA significantly suppressed the active state of Rac1, Cdc42, and RhoA with GTP status in U87 cells (Fig. 5c). Responsive to influence of ARHGDIA overexpression, the GTPase activity of Cdc42 and Rac1 was averagely decreased to 1.89- and 1.92-folds, and the GTPase activity of RhoA almost had almost no detection level (with average 6.8-folds decrease)(Fig. 5d). So we concluded that it has a significant role in the Rho GTPase signaling pathway by perturbations of ARHGDIA. Combined with the above conclusion of ARHGDIA downregulation in glioma cell migration, our studies confirmed that activation of signaling of Rho GTPases induced by silencing ARHGDIA contributes to cancer progression and metastasis for glioma.

### Downregulation of ARHGDIA increases Akt phosphorylation

**Fig. 6:**
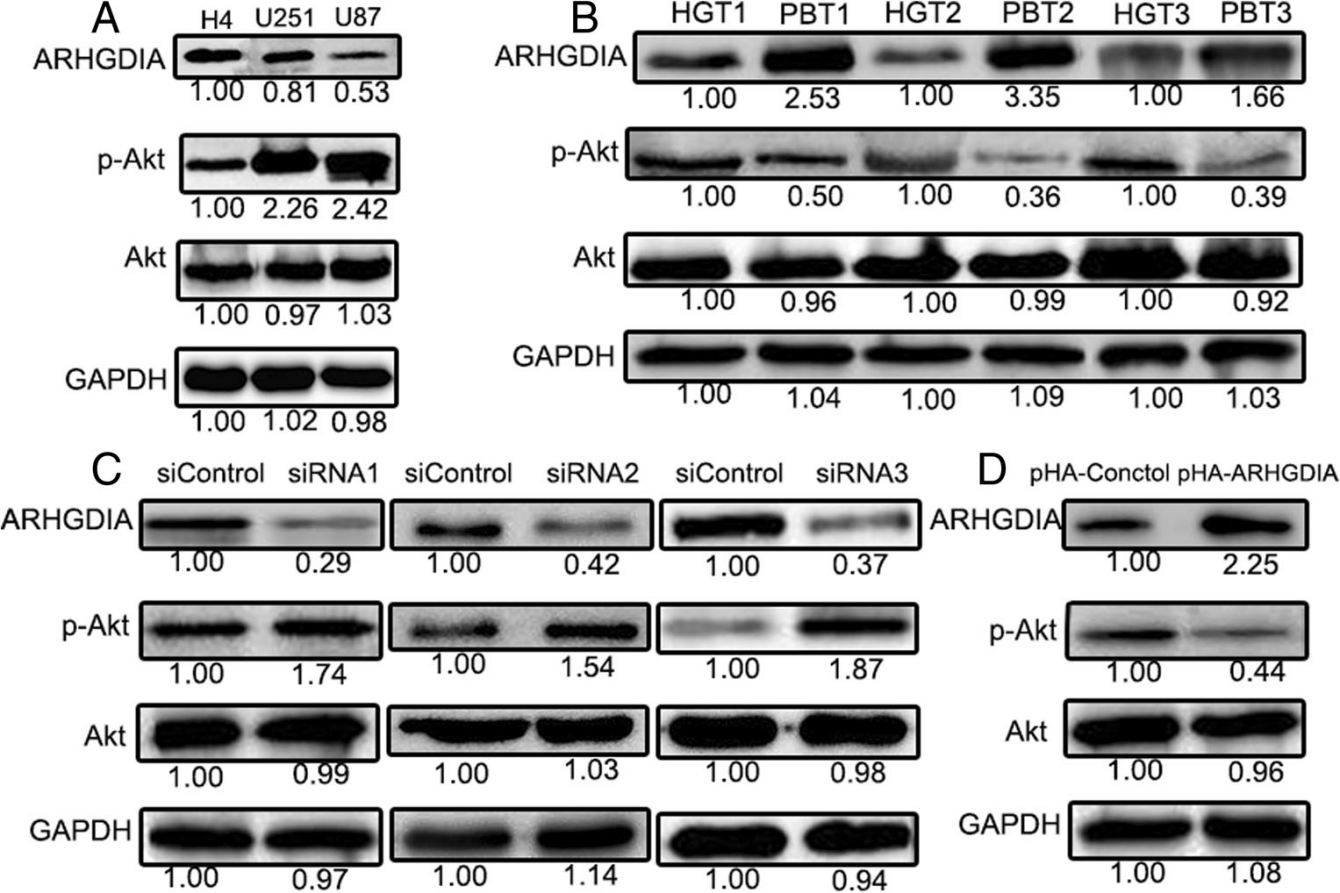
The Akt phosporylation level in human glioma cell lines (**a**, **c**) and in 3 randomly chosen HGTs (**b**, **d**). The 3 randomly chosen HGTs were the same samples used in the Fig. 1. The Akt phosporylation level was correlative with ARHGDIA expression in U87 cells and HGTs. siControl and pHA-control were the nontargeting control siRNAs or the empty transfection vector. pHA-ARHGDIA was ARHGDIA-overexpressing plasmid. *p-AKT *was the phosphorylated AKT

It had indicated that the GTPases of the Rho family control some major components of cellular signal transduction including Akt signaling, and inhibition of these GTPases leads to a decrease in Akt activity [15]. In this study, we also found that the phosphorylation of Akt (p-Akt) was high in human glioma cell lines, and the more higher degree of malignancy, the expression level of p-Akt was more higher (Fig. 6a). At the same time, we determined the expression level of p-Akt in HGTs and PBTs, the p-Akt expression was significantly increased in HGTs than PBTs (Fig. 6b). Combined with the ARHGDIA expression in HGTs, the above results suggest that the p-Akt expression may play an important role in the human glioma.

In order to explore whether the ARHGDIA expression had effects on p-Akt level, the phosphorylation level of Akt was further examined by Western blot after overexpression or knockdown of ARHGDIA in U87 cells. In order to reduce the off-target effects of single siRNA, we measured Akt phosphorylation changes responsive to ARHGDIA knockdown with three siRNAs (siRNA1, siRNA2, and siRNA3) treatment. The Akt phosphorylation was respectively upregulated to 1.63-, 1.78-, and 2.34-folds in U87 cells by the siRNA1, siRNA2, and siRNA3 incubation for 48 h compared with the nontargeting siRNA treatment ( Fig. 6c). On the contrary, Akt phosphorylation was decreased to 2.27-folds in ARHGDIA-overexpressing U87 cells (Fig. 6d). Therefore, the downregulation of ARHGDIA regulates GTPase activity of Cdc42, Rac1, and RhoA, subsequently, increases Akt phosphorylation and leads to glioma cell proliferation and migration.

## Discussion

Numerous studies indicate that GTPase signaling pathway closely correlates with tumors [16], and the expression of RhoGDIs which are the regulators of the Rho family of small GTPases is altered in different cancers. For instance, ARHGDIA expression is upregulated in colorectal [17] and ovarian cancers [18]. However, a significant reduction of ARHGDIA expression is detected in breast cancer [6]. In addition, ARHGDIA downregulation is associated with poor prognosis in hepatocellular carcinoma [7]. These different expression profiling even opposite results on this protein in several cancers urgently drives us to know how ARHGDIA mediates the processes during tumorigenesis and cancer progression in human glioma.

It has been reported ARHGDIA interacts with different proteins to involve in cell motility. ARHGDIA can interact with αvβ8 integrin, and ARHGDIA-αvβ8 integrin protein complexes recruit GDP-bound Rac1 and Cdc42 to control activation of Rho proteins. When silencing ARHGDIA gene expression leads to elevated levels of GTP-bound Rho proteins, which results in diminishing cell polarity and invasion [19]. Moreover, phosphorylation of ARHGDIA on Y156 leads to deposition of Rac1/Cdc42 proteins and enabling their activation to promote directional cell motility [20]. These indicate that the ARHGDIA protein affects the activation of Rho proteins mainly via coupling other proteins such as αvβ8 integrin. On the other hand, although ARHGDIA has been reported to decrease in brain tumors before [21], but there could not clearly clarify which member of RhoGDI family to change and its specific biological effects. In the present study, we further investigated the role of ARHGDIA in human glioma, and we firstly discovered the relationship between the dysregulation of ARHGDIA and glioma progression. Our research showed that ARHGDIA is frequently down-regulated in human glioma and significantly correlates with prognosis of glioma patients.

As the regulator of Rho GTPases family, we further focus on the changes of Rho GTPase proteins after ARHGDIA knockdown in human glioma cells. Previous researches had indicated that downregulation of ARHGDIA had a different effect on the activation of Rho GTPase family members in different tumor types. For example, Turner et al. found that the member of RhoGTPase was increased significantly in the HeLa cells transfected with ARHGDIA siRNA [22]. In myocardial cells, overexpression of ARHGDIA significantly inhibits the activities of Rac1, Cdc42, and RhoA [23]. But in ARHGDIA-knockout mice, renal abnormality is just associated with an increase of Rac1 but not RhoA [24], whereas loss of ARHGDIA significantly induced the activation of Rac1, RhoA but not Cdc42 in HCC [7]. By now, we found that knockdown of ARHGDIA by siRNA resulted in activating the GTPase activity of Cdc42, Rac1, RhoA, and pAkt to promote glioma cell proliferation and migration. To the best of our knowledge, this is the first study to discover the downregulation of ARHGDIA with glioma tumor progression by multiple biochemical analyses, overexpression, and knockdown in vitro combined with clinical sample validation.

**Fig. 7:**
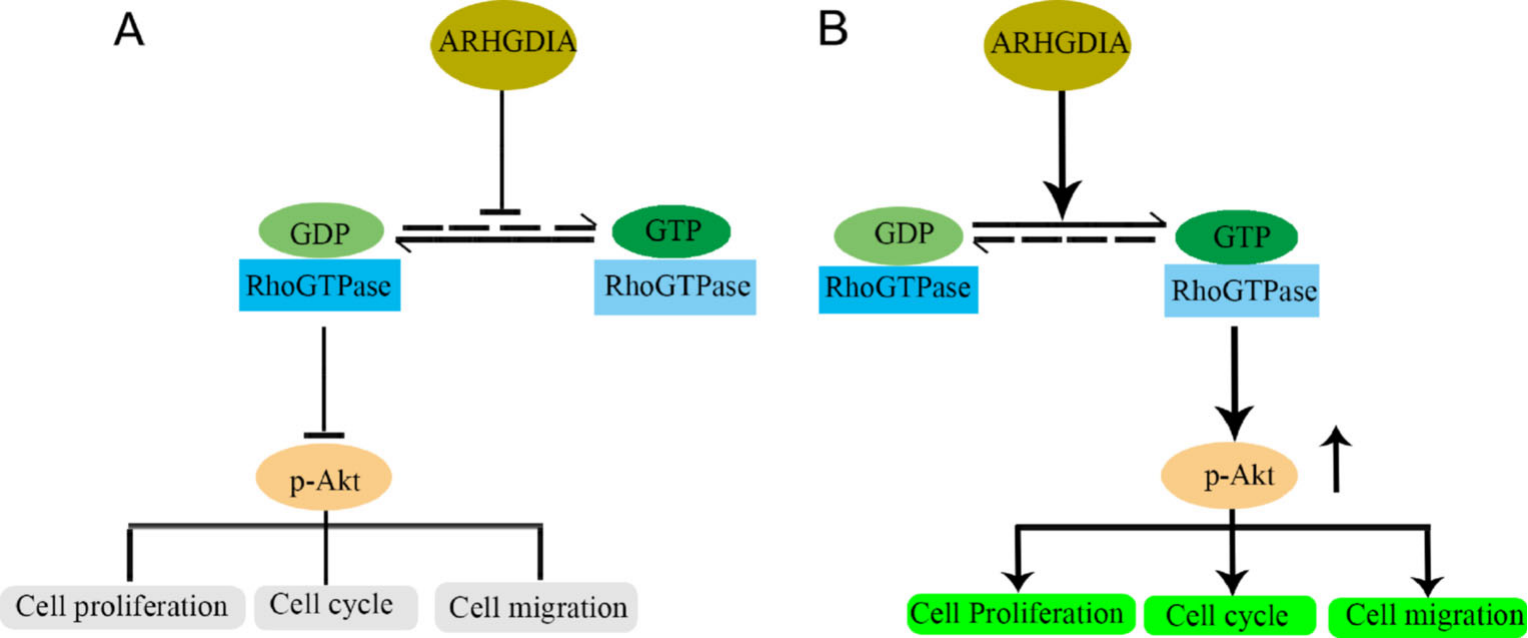
Schematic diagram summary of ARHGDIA effects on tumor progression in human gliomas. Under normal physiological conditions, ARHGDIA inhibits activation of Rho proteins by maintaining the GDP-bound Rho proteins in cytosol (**a**). When ARHGDIA protein is downregulated in glioma, the GTP-form Rho GTPases are activated (**b**) and subsequently Akt phosphorylaton is elevated, which promotes cell proliferation, cell cycle, and cell migration

Akt acts downstream of Rac1 and Cdc42 in the control of cellular survival [15], which indicates that Akt signaling is closely related to Rho GTPase signaling pathway. And our data supported that the activation of GTPase activity increases the downstream phosphorylated Akt signaling, which finally induces cell proliferation and cell cycle progression in human glioma cells. So based on the results of our research and literature reported, the potential action mechanism of ARHGDIA in glioma is summarized as following (Fig. 7). Under cell normal physiological conditions, ARHGDIA level makes a balance state of the GDP-bound inactive form and GTP-bound GTPase form of Rho family proteins (Fig. 7a). While the low expression of ARHGDIA in human glioma attenuates the inhibition effects of the GDP-bound inactive Rho family protein, and the GTP-bound form of Rho GTPases (Cdc42, Rac1, and Rho) is activated to switch on downstream pathways by acting on theirs effectors including improving phosphorylated Akt (Fig. 7b), which finally promotes cancer biological process including cell proliferation, cell cycle progression, and cell migration.

*ARHGDIA*, Rho GDP-dissociation inhibitor 1; *HGTs*: human glioma tissues; *IHC*, immunohistochemistry; *PBTs*, para-cancerous brain tissues; *siRNA*, small interfering RNA
